# Racial differences in primary sclerosing cholangitis: A retrospective cohort study leveraging a new ICD-10 code

**DOI:** 10.1016/j.aohep.2025.101901

**Published:** 2025-03-11

**Authors:** Melinda Wang, Alyssa Harris, Charles E. McCulloch, Sharad I. Wadhwani, Jennifer C. Lai, Jessica B. Rubin

**Affiliations:** a Department of Medicine, University of California-San Francisco, San Francisco, CA, United States; b Vizient, Inc., Chicago, IL, United States; c Department of Epidemiology and Biostatistics, University of California-San Francisco, San Francisco, CA, United States; d Department of Pediatrics, University of California-San Francisco, San Francisco, CA, United States; e Division of Gastroenterology and Hepatology, Department of Medicine, University of California-San Francisco, San Francisco, CA, United States

**Keywords:** PSC, Race, Disparity, Liver transplantation

## Abstract

**Introduction and Objectives:**

There are limited data examining racial disparities in PSC-related clinical features and events. We aimed to leverage a new PSC ICD-10 code to characterize racial differences in a nationwide contemporary cohort of patients with PSC.

**Patients and Methods:**

We used de-identified clinical/administrative data from the Vizient^®^ Clinical Data Base to identify outpatient and inpatient adult patients with PSC using a PSC-specific ICD-10-CM code (K83.01). We compared demographic and clinical characteristics between patients who self-identified as Black versus White race. Primary and secondary outcomes included clinical and healthcare utilization outcomes.

**Results:**

We identified 9850 patients with PSC: 79% White, 13% Black, 2% Asian, and 6% other. Compared with White patients, Black PSC patients were more likely to be female, younger, and have a higher Elixhauser Comorbidity Index (p<0.001). Black patients were less likely to have concurrent inflammatory bowel disease (IBD) but more likely to have overlap syndromes (p<0.001) compared to White patients. The black race was significantly associated with intensive care unit stays (aOR 1.69, 95%CI 1.38–2.06) and longer length of stay (aIRR 1.15, 95%CI 1.04–1.28), but similar total number of hospitalizations (aIRR 1.05, 95%CI 0.98–1.13) compared to the White race.

**Conclusions:**

Black patients had sociodemographic and clinical features suggestive of more severe disease compared to White patients. Although hospitalization rates were similar, Black patients received higher-intensity care. There was no significant difference in liver transplantation between Black and White patients. Additional research on optimal diagnosis and management of PSC in Black populations specifically is necessary to reduce healthcare disparities in this disease.

## 1. Introduction

Primary sclerosing cholangitis (PSC) is a progressive chronic liver disease associated with inflammation of the biliary tree. It is often associated with high healthcare utilization and there are few therapeutic options short of liver transplantation [1]. As the most common demographic of patients typically affected by PSC have been White males, research on PSC has traditionally focused on this study population [2]. Few studies have explored racial diversity among patients with PSC [3].

Racial disparities are persistent in GI and liver diseases, though there have been limited data on disparities in PSC specifically. A few studies have looked specifically at racial disparities among patients with PSC awaiting liver transplant in the US, finding that Black patients with PSC tend to be younger and present to transplant centers with more severe disease [4]. However, it is unclear whether racial disparities in disease presentation and outcomes exist at earlier stages of disease, or among those not referred for liver transplant [3].

Previous PSC research has been limited to small, single-centered studies or national and international registries [2,5−9]. A new PSC-specific ICD-10 code was introduced in 2018, which allows for more accurate identification of patients with PSC in administrative databases. We previously demonstrated successful use of this PSC-specific ICD-10 code (K83.01) to identify hospitalized patients with PSC in a large administrative database [10]. In the present study, we aimed to further leverage this ICD-10 code to more thoroughly characterize racial disparities in PSC-related complications and healthcare utilization outcomes among these patients in a large, nationwide administrative database.

## 2. Patients and Methods

Data were collected from de-identified clinical and administrative data from the Vizient Clinical Data Base used with permission of Vizient, Inc. All rights reserved. The Vizient Clinical Data Base is a performance improvement database including administrative data from >1000 academic, affiliated, and community hospitals nationwide. Adult patients ≥18 years old who had either: (1) ≥ 2 inpatient or outpatient encounters with the PSC-specific ICD-10 code (K83.01) from 10/1/2018 to 12/31/2021; or (2) 1 encounter with the PSC-specific ICD-10 code from 10/1/2018–12/31/2021 and ≥ 1 encounter with cholangitis ICD-9 code (576.1) from 10/1/2018–12/31/2021 were included in the study.

Socioeconomic factors, including age, sex, insurance, and region, were available in the database. We used inpatient and outpatient ICD-10 codes to identify comorbidities and PSC-related clinical factors including concurrent diagnoses of: inflammatory bowel disease, primary biliary cholangitis, autoimmune hepatitis, cirrhosis, hepatocellular carcinoma, and cholangiocarcinoma were also collected (Supplemental Table 1). ICD-10 codes were also used to calculate Elixhauser Comorbidity Index. The Elixhauser Comorbidity Index was chosen due to its ability to accommodate both inpatient and outpatient data [11]. PSC-related complications included development of cirrhosis and admissions for sepsis/biliary obstruction, cirrhosis, and cancer (e.g. hepatocellular carcinoma, cholangiocarcinoma). Laboratory values were calculated as a median of all outpatient laboratory values available in the database. Our healthcare utilization outcomes included: number of hospitalizations, hospital length of stay, and intensive care unit admissions,

Given small numbers of non-White, non-Black patients in our cohort, our primary analyses focused on comparisons in demographics and outcomes between Black and White patients with PSC. Demographic and clinical characteristics were compared between Black and White patients with PSC using chi-squared testing or Wilcoxon rank sum analysis based on dichotomous or continuous variable characteristics. Multivariable analyses using Poisson or logistic regression were used for count-associated and binary outcomes, respectively. Quarterly data, but not data by days or years, were available for this study. Therefore, for time-dependent outcomes, follow-up time was accounted for by using the total number of quarters a patient was followed as an offset. Covariates included in multivariable models were determined through a combination of a priori identification of related sociodemographic variables that were identified in previous literature [10] as clinically significant, sample size, and by backwards selection for variables with a significance defined as p-value <0.01. A p-value <0.05 was considered statistically significant. Data were analyzed using Stata/MP 16.1 statistical software (College Station, TX).

### 2.1. Ethical statement

This study was approved by the Institutional Review Board at University of California San Francisco (21–35754).

## 3. Results

**Fig. 1.**
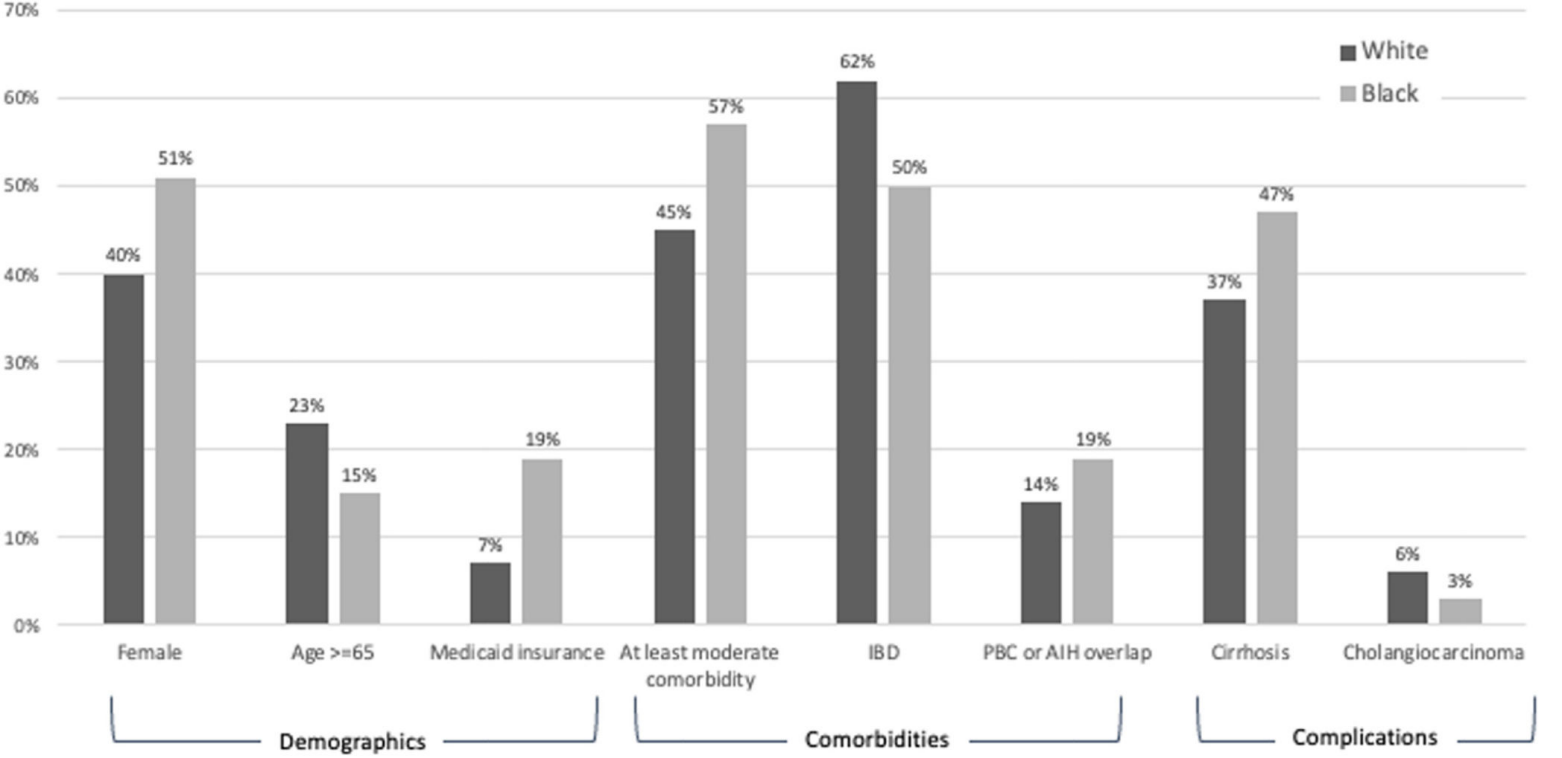
Demographic and clinical differences between black and white patients with PSC.

Of 9850 patients with PSC between 10/1/2018 and 12/31/2021, 7245 (79%) were White, 1185 (13%) were Black, 211 (2%) were Asian, 586 (6%) identified as Other race, and 623 (6%) had missing race. 42% of the cohort was female and median age was 49 (IQR 35–63); 59% had commercial insurance; 38% had cirrhosis. Compared to White patients with PSC, Black patients with PSC were more likely to be female and younger and less likely to have commercial insurance (Table 1, Fig. 1). Black patients were also more likely to live in the South compared to White patients.

Regarding clinical characteristics, Black patients had higher Elixhauser Comorbidity Index compared with White patients. Black patients were more likely to have a diagnosis of both PSC and autoimmune liver disease (AIH: 9% vs 6%, p<0.001; PBC: 10% vs 8%, p=0.07) but less likely to have inflammatory bowel disease (50% vs 62%, p < 0.001) than White patients (Table 1, Fig. 1). Regarding PSC complications, Black patients seem to have more severe disease characterized by higher median alkaline phosphatase [244 (IQR 131–483) vs 174 (IQR 103–329), p<0.001], higher rates of cirrhosis (47% vs 37%, p<0.001), and higher MELD-Na scores among patients with cirrhosis [9 (IQR 6–14) vs 8 (5–13), p = 0.003]. Rates of decompensation were similar between Black and White patients with cirrhosis (38% vs 41%, p=0.17). Although White patients were more likely to have cholangiocarcinoma (6% vs 3%, p=0.006), there was no significant difference in hepatocellular carcinoma between Black and White patients (2% for both, p = 0.88).

### 3.1. Clinical outcomes

**Fig. 2.**
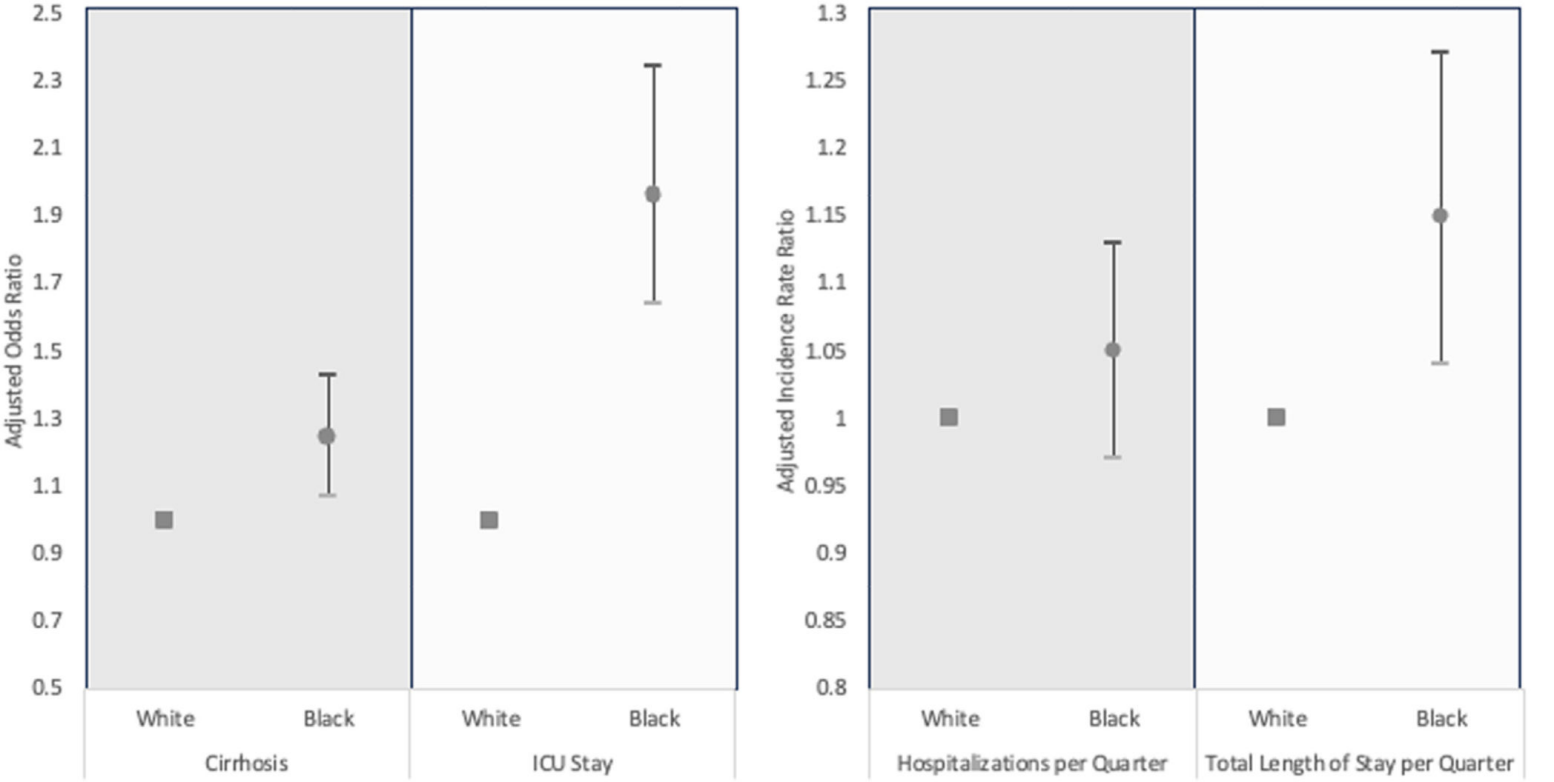
Clinical outcomes and healthcare utilization differences between black and white patients with PSC.

On logistic regression, Black race was associated with increased odds of cirrhosis (OR 1.49, 95%CI 1.30–1.70, p<0.001). Other factors associated with cirrhosis diagnosis included gender, insurance, region, Elixhauser Comorbidity Index, and concurrent diagnosis of PSC and autoimmune hepatitis. On multivariable regression, after adjustment for these variables, Black race remained significantly associated with cirrhosis diagnosis (aOR 1.20, 95%CI 1.01–1.43, p=0.04) (Table 2, Fig. 2). There was no significant difference in liver transplantation rates (20% vs 20%, p=0.42) between Black and White patients during the observed study period.

### 3.2. Healthcare utilization

There was no significant difference in number of outpatient ambulatory visits per quarter in Black versus White patients (3 (IQR 2–3) vs 3 (IQR 2–3), p = 0.63); however, Black race was associated with a 13% increased rate of number of hospitalizations (IRR 1.13, 95%CI 1.05–1.22) (Table 3). Other factors associated with hospitalization included: gender, age ≥ 65 years old, primary insurance, Elixhauser comorbidity index, and presence of IBD. However, on multivariable regression, after adjustment for sociodemographic, Elixhauser comorbidity index, and concurrent IBD diagnosis, the Black race was no longer associated with the risk of hospitalization (aIRR 1.05, 95%CI 0.98–1.13) (Table 3). While reasons for hospitalization were similar between Black and White patients (Supplemental Table 2), Black patients had longer hospital length of stay (IRR 1.17, 95%CI 1.06–1.29), and were more likely to require intensive care unit admission (OR 2.74, 95%CI 2.35–4.21). These differences persisted on multivariable analysis after adjustment for demographic and clinical factors (length of stay: aIRR 1.15, 95%CI 1.04–1.28; intensive care unit admission: aOR 1.69, 95%CI 1.38–2.06) (Tables 4 and 5, Fig. 2).

## 4. Discussion

Given the classical White predominance among patients with PSC, prior studies of the disease have historically focused on this population [1]. With the release of a new ICD-10 code specific for patients with PSC in 2018, administrative datasets can now be used to more effectively identify and study patients with PSC without the limitations of single center studies [3,12]. This study leverages the new PSC-specific code within a contemporary nationwide database to identify those with PSC across clinical settings, and to characterize racial disparities in clinical and healthcare utilization outcomes among this PSC cohort while adjusting for covariates.

We observed that among patients with PSC, Black patients tend to have more severe disease—as characterized by higher median alkaline phosphatase values, increased rates of cirrhosis, and higher median MELD-Na among those with cirrhosis—than White patients with PSC. This data is consistent with prior small single-center studies, as well as our prior study of hospitalized patients with PSC during the same time period [1,3,4,10]. In addition, we found that Black race was associated with lower rates of concurrent IBD, but increased rates of overlapping autoimmune liver disease. Despite these differences, rates of liver transplant were similar. While this discrepancy between may be due to the cross-sectional nature of the dataset, socioeconomic disparities to access to liver transplantation is well established in the literature [13,14]. Whether this discrepancy between severity of liver disease and transplantation rates are due to limitations within the dataset versus disparities in liver transplantation would require additional evaluation. As with the prior hospitalized PSC patient cohort, we identify racial disparities between Black and White patients among hospital length of stay and ICU stay [10]. Black race was not associated with number of hospitalizations on simple, but not adjusted analysis [10].

What might explain our observed racial differences among Black and White patients with PSC? Previous studies have suggested that these differences may in part be attributed to socioeconomic factors [2]. While large administrative datasets may not have available variables to adequately assess differences in specific socioeconomic factors, our study suggest that observed racial differences are due to more than just differences in comorbidity between Black and White patients, including hospital setting. Race is a social construct, and measuring the effects of societal racism and racial disparities are a result of structural and interpersonal racism. Some of our findings, such as increased rates of cirrhosis among Black patients, may be explained by the downstream effects of racism in this population—whether it be decreased access to tertiary care centers, delayed diagnosis, or differences in management. Prior studies have suggested that, in fact, Black patients do often present at a later stage of disease compared to White patients [15,16]. Compared to the classic PSC patient who is diagnosed in the setting of known inflammatory bowel disease, Black PSC patients—who are less likely to have a concurrent inflammatory bowel disease diagnosis—may experience delayed initial recognition of disease or they may have a missed diagnosis of inflammatory bowel disease [17]. Black patients’ increased rates of overlapping autoimmune liver diseases may further confound diagnosis in these patients. Liver transplant rates, however, are similar between Black and White patients. Whether comparable rates of liver transplantation despite evidence of more severe disease among Black patients is due to racial disparities remains to be explored.

Despite its novelty, this study has some limitations. First, delays in implementation of the ICD-10 code across hospital systems may lead to underestimation of the PSC cohort in this study. Since the inclusion criteria included at least 2 ICD-10 codes in the inpatient or outpatient setting or 1 ICD-9 code for cholangitis and 1 ICD-10 code for PSC, we may also miss patients with PSC who may have only had one encounter in this administrative dataset. Second, the large sample size afforded adjusting for covariates that may partially explain the observed racial differences, but we were limited by other individual and neighborhood related variables in this dataset to explore additional mediators that may explain racial disparities in this study or severity of PSC. Third, data were limited to encounters within hospitals that are Vizient customers participating in the Vizient Clinical Data Base. Patients with ambulatory and hospital encounters outside of hospitals captured by the Vizient database, representing potential fragmentation of care, were not available in this database.

## 5. Conclusions

Nonetheless, our data demonstrate the utility of using this new ICD-10 code to identify large cohorts of PSC patients across clinical settings. In our prior work, we identified 944 inpatients with PSC, in the present study, we identified approximately 10-fold more PSC patients in the same time period [10]. The similarity of findings between this study and prior single center studies examining racial differences in PSC suggest that this ICD-10 code is a valid tool for identifying patients with PSC in large administrative databases. Importantly, the large sample size has allowed for better characterization of racial disparities throughout a patient’s PSC clinical course, as well as clinical and healthcare utilization outcomes among patients with PSC. These findings should better assist with examining mediators of racial disparities among patients with PSC and identifying effective interventions to reduce healthcare disparities among patients with PSC.

## Supplementary Material

Supp 3

Supp 1

Supp 2

Supp 4

Supp 5

Supplementary material associated with this article can be found in the online version at doi:10.1016/j.aohep.2025.101901.

## Figures and Tables

**Table 1:** Baseline characteristics and encounters for patients with PSC by white vs black race.

Factor	White (n=7245, 86%)	Black (n=1185, 14%)	p-value

*Demographics*			
Female	2875 (40)	607 (51)	<0.001
Age ≥ 65	1644 (23)	178 (15)	<0.001
Hispanic	241 (4)	17 (2)	<0.001
Primary Payer			<0.001
Commercial	4475 (62)	545 (46)	
Medicaid	479 (7)	223 (19)	
Medicare	1965 (27)	335 (28)	
Other	326 (5)	82 (7)	
Secondary Payer			0.01
Commercial	1949 (27)	273 (23)	
Other	5306 (73)	912 (77)	
Region			<0.001
Midwest	2760 (38)	365 (31)	
Northeast	1010 (14)	136 (12)	
South	1548 (21)	529 (45)	
West	1927 (27)	155 (13)	
*Laboratory Values*			
Median Alkaline phosphatase	174 (103–329)	244 (131–483)	<0.001
Median Total bilirubin	0.8 (0.5–1.5)	0.9 (0.5–2.2)	<0.001
Median platelet	222 (152–290)	231 (164–299)	0.11
Median MELD-Na (among patients with cirrhosis)	8 (5–13)	9 (6–14)	0.003
Median INR (among patients with cirrhosis)	1.15 (1.00–1.30)	1.19 (1.06–1.40)	0.03
*Comorbidities/Complications*			
Elixhauser Comorbidity Index, mean (SD)	2.6 (3.3)	3.3 (2.6)	<0.001
Sepsis	41 (12)	8 (10)	0.62
Cholangitis	4203 (68)	702 (67)	0.59
Pancreatitis	287 (5)	43 (4)	0.45
PSC-primary biliary cholangitis Overlap	610 (8)	119 (10)	0.07
PSC-autoimmune hepatitis Overlap	424 (6)	102 (9)	<0.001
Cirrhosis	2281 (37)	486 (47)	<0.001
Decompensated Cirrhosis (among patients with cirrhosis)	946 (41)	185 (38)	0.17
Hepatocellular carcinoma	123 (2)	20 (2)	0.87
Cholangiocarcinoma	340 (6)	36 (3)	0.006
Inflammatory bowel disease	3825 (62)	521 (50)	<0.001
Ulcerative Colitis	2787 (45)	371 (36)	<0.001
Crohn’s Disease	1236 (20)	180 (17)	0.04

Data from the Vizient Clinical Data Base used with permission of Vizient, Inc. All rights reserved. INR, International Normalized Ratio; MELD-Na, Mayo End-stage Liver Disease-Sodium; SD, Standard Deviation.

**Table 2:** Simple and adjusted logistic regression for cirrhosis.

	Simple			Adjusted		
	OR	95%CI	p-value	OR	95%CI	p-value

Female	0.87	0.79–0.95	0.004	0.71	0.62–0.80	<0.001
Black	1.49	1.30–1.70	<0.001	1.20	1.01–1.43	0.04
Age ≥65	1.24	1.11–1.39	<0.001	0.93	0.76–1.15	0.51
Hispanic	1.21	0.92–1.60	0.17			
Primary Payer						
Commercial	Ref	Ref	Ref	Ref	Ref	Ref
Medicaid	1.57	1.32–1.87	<0.001	1.25	1.01–1.55	0.04
Medicare	1.55	1.39–1.72	<0.001	0.91	0.75–1.11	0.35
Other	1.18	0.94–1.48	0.153	1.05	0.79–1.39	0.74
Region						
Midwest	Ref	Ref	Ref	Ref	Ref	Ref
Northeast	1.67	1.01–1.35	0.04	1.10	0.92–1.33	0.30
South	1.16	1.03–1.32	0.02	1.10	0.94–1.29	0.22
West	0.90	0.79–1.01	0.09	0.94	0.81–1.11	0.47
Elixhauser Comorbdity Index	1.52	1.48–1.59	<0.001	1.55	1.51–1.59	<0.001
Inflammatory bowel disease	1.06	0.96–1.17	0.23	0.95	0.84–1.08	0.42
PSC-primary biliary cholangitis overlap	1.00	−	−	1	−	−
PSC-autoimmune hepatitis overlap	2.54	2.12–3.05	<0.001	2.91	2.33–3.63	<0.001

Data from the Vizient Clinical Data Base used with permission of Vizient, Inc. All rights reserved.

**Table 3:** Simple and adjusted poisson regression for total number of hospitalizations.

	Simple			Adjusted		
	IRR	95%CI	p-value	aIRR	95%CI	p-value

Female	0.93	0.88–0.98	0.01	0.91	0.86–0.96	0.001
Black	1.13	1.05–1.22	0.001	1.05	0.98–1.13	0.20
Age ≥65	0.88	0.83–0.94	<0.001	0.76	0.69–0.84	<0.001
Hispanic	1.08	0.94–1.25	0.26	1.04	0.92–1.19	0.52
Region						
Midwest	Ref	Ref	Ref			
Northeast	1.01	0.94–1.10	0.73			
South	0.97	0.90–1.04	0.38			
West	0.93	0.86–1.00	0.05			
Primary Payer						
Commercial	Ref	Ref	Ref	Ref	Ref	Ref
Medicaid	1.36	1.24–1.49	<0.001	1.22	1.11–1.34	<0.001
Medicare	1.13	1.06–1.20	<0.001	1.06	0.96–1.16	0.24
Other	1.24	1.09–1.42	0.002	1.20	1.04–1.38	0.01
Elixhauser Comorbidity Index	1.14	1.13–1.15	<0.001	1.15	1.14–1.16	<0.001
Inflammatory bowel disease	1.11	1.04–1.17	0.001	1.09	1.03–1.16	0.01

Data from the Vizient Clinical Data Base used with permission of Vizient, Inc. All rights reserved. IRR, Incidence Rate Ratio.

**Table 4:** Simple and adjusted poisson regression for LOS per quarter.

	Simple			Adjusted		
	IRR	95%CI	p-value	aIRR	95%CI	p-value

Female	1.00	0.93–1.08	0.98			
Black	1.17	1.06–1.29	0.002	1.15	1.04–1.28	0.01
Age ≥65	1.03	0.94–1.13	0.65			
Hispanic	1.38	1.16–1.64	<0.001	1.42	1.18–1.70	<0.001
Region						
Midwest	Ref	Ref	Ref			
Northeast	0.89	0.80–0.99	0.04			
South	1.05	0.95–1.17	0.30			
West	1.06	0.96–1.16	0.23			
Primary Payer						
Commercial	Ref	Ref	Ref	Ref	Ref	Ref
Medicaid	1.16	1.02–1.31	0.02	1.02	0.90–1.16	0.74
Medicare	1.15	1.06–1.25	0.001	0.96	0.87–1.106	0.38
Other	0.99	0.82–1.19	0.91	0.96	0.79–1.17	0.69
Elixhauser Comorbidity Index	1.13	1.12–1.14	<0.001	1.13	1.12–1.15	<0.001
Inflammatory bowel disease	1.17	1.08–1.26	<0.001	1.22	1.12–1.32	<0.001

Data from the Vizient Clinical Data Base used with permission of Vizient, Inc. All rights reserved. aIRR, Adjusted Incidence Rate Ratio; IRR, Incidence Rate Ratio.

**Table 5:** Simple and adjusted logistic regression for ICU stay.

	Simple			Adjusted		
	OR	95%CI	p-value	OR	95%CI	p-value

Female	0.73	0.66–0.82	<0.001	0.62	0.54–0.72	<0.001
Black	2.74	2.35–4.21	<0.001	1.69	1.38–2.06	<0.001
Age ≥65	1.09	0.96–1.24	0.17			
Hispanic	0.73	0.52–1.02	0.07	0.80	0.53–1.22	0.30
Region						
Midwest	Ref	Ref	Ref	Ref	Ref	Ref
Northeast	0.90	0.76–1.06	0.22	0.85	0.68–1.06	0.14
South	0.86	0.74–0.98	0.03	0.77	0.65–0.93	0.005
West	0.68	0.59–0.79	<0.001	0.70	0.58–0.84	<0.001
Primary Payer						
Commercial	Ref	Ref	Ref	Ref	Ref	Ref
Medicaid	1.53	1.27–1.85	<0.001	0.81	0.63–1.04	0.10
Medicare	1.49	1.32–1.68	<0.001	0.61	0.52–0.72	<0.001
Other	1.21	0.94–1.56	0.142	0.89	0.63–1.26	0.50
Elixhauser Comorbidity Index	1.65	1.60–1.69	<0.001	1.68	1.63–1.74	<0.001
Inflammatory bowel disease	1.27	1.14–1.43	<0.001	1.06	0.91–1.23	0.45

Data from the Vizient Clinical Data Base used with permission of Vizient, Inc. All rights reserved. CI, Confidence Interval; OR, Odds Ratio.

## Abbreviations:

aIRR: adjusted incidence rate ratio
aOR: adjusted odds ratio
ICD-9: internal classification of disease, 9th revision − clinical modification
ICD-10: internal classification of disease, 10th revision − clinical modification
MELD-Na: mayo end-stage liver disease-sodium
PSC: primary sclerosing cholangitis
