# Supplementary material for: Racial differences in primary sclerosing cholangitis: A retrospective cohort study leveraging a new ICD-10 code
Source: Ann Hepatol. Author manuscript; Available in PMC 2026 Mar 11. (PMC12826390; doi:10.1016/j.aohep.2025.101901)
Supplement: Supp 3 [file NIHMS2089701-supplement-Supp_3.docx]

**Supplemental Table 4. Logistic Regression for early ERCP**

| Variables | Univariable |  |  | Multivariable |  |  |
| --- | --- | --- | --- | --- | --- | --- |
|  | OR | 95%CI | p-value | aOR | 95%CI | p-value |
| Race / Ethnicity  White  Black  Hispanic  Other | --  0.77  0.92  0.92 | --  0.66-0.89  0.81-1.04  0.80-1.05 | --  0.001  0.192  0.217 | --  0.75  0.99  0.90 | --  0.64-0.88  0.86-1.13  0.78-1.03 | --  <0.001  0.835  0.158 |
| Age ≥ 65 | 1.20 | 1.11-1.29 | <0.001 | 1.26 | 1.16-1.37 | <0.001 |
| Female | 0.98 | 0.92-1.06 | 0.651 | 1.02 | 0.94-1.10 | 0.600 |
| Insurance  Private  Public  Other | --  0.94  0.94 | --  0.87-1.02  0.80-1.10 | --  0.155  0.442 |  |  |  |
| Income  Quartile 1  Quartile 2  Quartile 3  Quartile 4 | --  0.98  1.02  1.08 | --  0.88-1.10  0.91-1.13  0.97-1.20 | --  0.743  0.765  0.140 |  |  |  |
| Charlson Severity Index  Mild  Moderate  Severe | --  1.17  1.43 | --  1.07-1.27  1.29-1.58 | --  0.001  <0.001 | --  0.87  0.74 | --  0.79-0.96  0.65-0.85 | --  0.006  <0.001 |
| Sepsis | 0.86 | 0.76-0.97 | 0.015 | 0.95 | 0.82-1.09 | 0.442 |
| Bacteremia | 0.90 | 0.84-0.98 | 0.011 | 0.86 | 0.79-0.95 | 0.002 |
| Pancreatitis | 0.78 | 0.70-0.88 | <0.001 | 1.01 | 0.90-1.15 | 0.833 |
| Gallstone disease | 0.58 | 0.54-0.63 | <0.001 | 0.72 | 0.65-0.79 | <0.001 |
| Liver/biliary/pancreatic malignancy | 2.61 | 2.40-2.84 | <0.001 | 2.52 | 2.23-2.80 | <0.001 |
| Hospital type  Rural  Urban non-teaching  Urban teaching | --  1.54  1.92 | --  1.30-1.84  1.62-2.27 | --  <0.001  <0.001 | --  1.48  1.72 | --  1.23-1.79  1.43-2.07 | --  <0.001  <0.001 |
| Region  Northeast  Midwest  South  West | --  0.66  0.73  0.85 | --  0.59-0.74  0.66-0.81  0.77-0.94 | --  <0.001  <0.001  0.002 | --  0.97  0.81  0.89 | --  0.86-1.09  0.73-0.90  0.80-1.00 | --  0.619  <0.001  0.043 |
