# Supplementary material for: Racial differences in primary sclerosing cholangitis: A retrospective cohort study leveraging a new ICD-10 code
Source: Ann Hepatol. Author manuscript; Available in PMC 2026 Mar 11. (PMC12826390; doi:10.1016/j.aohep.2025.101901)
Supplement: Supp 1 [file NIHMS2089701-supplement-Supp_1.docx]

**Supplemental Table 5. Zero-Adjusted Poisson Regression for Hospital Length of Stay**

| Variables | Univariable |  |  | Multivariable |  |  |
| --- | --- | --- | --- | --- | --- | --- |
|  | IRR | 95%CI | p-value | Adjusted IRR | 95%CI | p-value |
| Race / Ethnicity  White  Black  Hispanic  Other | --  1.29  1.10  1.14 | --  1.26-1.32  1.08-1.13  1.12-1.17 | --  <0.001  <0.001  <0.001 | --  1.22  1.09  1.09 | --  1.19-1.25  1.06-1.11  1.07-1.12 | --  <0.001  <0.001  <0.001 |
| Age ≥ 65 | 1.00 | 0.99-1.01 | 0.933 |  |  |  |
| Female | 0.96 | 0.95-0.98 | <0.001 | 0.98 | 0.97-0.99 | 0.002 |
| Insurance  Private  Public  Other | --  0.89  0.94 | --  0.87-0.90  0.91-0.96 | --  <0.001  <0.001 | --  0.95  0.99 | --  0.93-0.96  0.96-1.02 | --  <0.001  0.580 |
| Income  Quartile 1  Quartile 2  Quartile 3  Quartile 4 | --  0.93  0.93  0.94 | --  0.92-0.95  0.92-0.95  0.92-0.96 | --  <0.001  <0.001  <0.001 |  |  |  |
| Charlson Severity Index  Mild  Moderate  Severe | --  1.17  1.34 | --  1.15-1.19  1.31-1.36 | --  <0.001  <0.001 | --  1.14  1.24 | --  1.12-1.16  1.21-1.26 | --  <0.001  <0.001 |
| Sepsis | 1.69 | 1.67-1.72 | <0.001 | 1.58 | 1.55-1.61 | <0.001 |
| Bacteremia | 1.52 | 1.50-1.54 | <0.001 | 1.27 | 1.25-1.29 | <0.001 |
| Pancreatitis | 1.23 | 1.20-1.25 | <0.001 | 1.24 | 1.21-1.26 | <0.001 |
| Gallstone disease | 1.02 | 1.01-1.03 | 0.004 | 0.99 | 0.97-1.00 | 0.073 |
| Liver/biliary/pancreatic malignancy | 1.07 | 1.05-1.08 | <0.001 | 0.99 | 0.97-1.01 | 0.217 |
| Hospital type  Rural  Urban non-teaching  Urban teaching | --  1.33  1.51 | --  1.30-1.37  1.48-1.55 | --  <0.001  <0.001 | --  1.27  1.46 | --  1.24-1.31  1.42-1.50 | --  <0.001  <0.001 |
| Region  Northeast  Midwest  South  West | --  -.85  0.96  0.89 | --  0.83-0.86  0.94-0.98  0.87-0.91 | --  <0.001  <0.001  <0.001 | --  0.88  0.98  0.89 | --  0.86-0.90  0.96-1.00  0.87-0.91 | --  <0.001  0.028  <0.001 |
| Early ERCP | 0.77 | 0.76-0.79 | <0.001 | 0.78 | 0.76-0.79 | <0.001 |

ERCP ,endoscopic retrograde cholangiopancreatography
