# Supplementary material for: Racial differences in primary sclerosing cholangitis: A retrospective cohort study leveraging a new ICD-10 code
Source: Ann Hepatol. Author manuscript; Available in PMC 2026 Mar 11. (PMC12826390; doi:10.1016/j.aohep.2025.101901)
Supplement: Supp 2 [file NIHMS2089701-supplement-Supp_2.docx]

**Supplemental Table 2.** Procedure ICD codes

| Diagnosis | ICD-9 Code | ICD-10 Code |
| --- | --- | --- |
| Diagnostic/Therapeutic ERCP | 518.4, 518.5, 518.6, 518.7, 518.8, 511.1, 521.3, 511,0, 511.4, 521.4, 516.4, 522.1, 511.5 | 0FJB8ZZ, 0FJD8ZZ, 0F9C8ZZ, 0F758DZ, 0F768DZ, 0F788DZ, 0F798DZ, 0F7C8DZ, 0F9580Z, 0F9680Z, 0F9880Z, 0F9C80Z, 0FC58ZZ, 0FC68ZZ, 0FC88ZZ, 0Fc98ZZ, 0FCC8ZZ, 0FF58ZZ, 0FF68ZZ, 0FF88ZZ, 0FF98ZZ, 0FFC8ZZ, 0FJD8ZZ, 0F7D8DZ, 0F7F8DZ, 0F9D80Z, 0F9F80Z, 0FCD8ZZ, 0FCF8ZZ, 0FFD8ZZ, 0FFF8ZZ |
| Cholecystectomy | 51.2, 51.21, 51.22, 51.23, 51.24 | 0FT44ZZ, 0FB44ZZ, 0FT50ZZ, 0FB40ZZ |
| Percutaneous Biliary Drainage | 51.98, 51.96 | 0F9430Z, 0F943ZX, 0F943ZZ, 0F9440Z, 0F944ZX, 0F944ZZ |
