# Supplementary material for: Racial differences in primary sclerosing cholangitis: A retrospective cohort study leveraging a new ICD-10 code
Source: Ann Hepatol. Author manuscript; Available in PMC 2026 Mar 11. (PMC12826390; doi:10.1016/j.aohep.2025.101901)
Supplement: Supp 4 [file NIHMS2089701-supplement-Supp_4.docx]

**Supplemental Table 3. Logistic Regression for ERCP**

| Variables | Univariable |  |  | Multivariable |  |  |
| --- | --- | --- | --- | --- | --- | --- |
|  | OR | 95%CI | p-value | aOR | 95%CI | p-value |
| Race / Ethnicity  White  Black  Hispanic  Other | --  0.70  1.31  1.26 | --  0.66-0.75  1.23-1.39  1.18-1.34 | --  <0.001  <0.001  <0.001 | --  0.78  1.18  1.15 | --  0.73-0.85  1.10-1.26  1.07-1.24 | --  <0.001  <0.001  <0.001 |
| Age ≥ 65 | 1.64 | 1.59-1.70 | <0.001 | 1.36 | 1.29-1.44 | <0.001 |
| Female | 1.10 | 1.06-1.13 | <0.001 | 1.07 | 1.03-1.12 | 0.001 |
| Insurance  Private  Public  Other | --  0.70  1.02 | --  0.67-0.73  0.95-1.09 | --  <0.001  0.639 | --  0.95  1.19 | --  0.90-1.01  1.18-1.30 | --  0.077  <0.001 |
| Income  Quartile 1  Quartile 2  Quartile 3  Quartile 4 | --  1.02  1.11  1.11 | --  0.97-1.07  1.05-1.16  1.06-1.17 | --  0.477  <0.001  <0.001 |  |  |  |
| Charlson Severity Index  Mild  Moderate  Severe | --  1.02  0.77 | --  0.98-1.06  0.73-0.81 | --  0.278  <0.001 | --  0.93  0.85 | --  0.89-0.98  0.80-0.91 | --  0.003  <0.001 |
| Sepsis | 0.93 | 0.88-0.98 | 0.009 | 0.77 | 0.72-0.83 | <0.001 |
| Bacteremia | 1.11 | 1.07-1.15 | <0.001 | 1.08 | 1.03-1.14 | 0.001 |
| Pancreatitis | 1.96 | 1.98-2.06 | <0.001 | 1.50 | 1.41-1.59 | <0.001 |
| Gallstone disease | 4.42 | 4.43-4.46 | <0.001 |  |  |  |
| Liver/biliary/pancreatic malignancy | 0.86 | 0.82-0.90 | <0.001 |  |  |  |
| Hospital type  Rural  Urban non-teaching  Urban teaching | --  2.47  2.11 | --  2.31-2.65  1.97-2.25 | --  <0.001  <0.001 | --  2.41  2.68 | --  2.21-2.62  2.46-2.01 | --  <0.001  <0.001 |
| Region  Northeast  Midwest  South  West | --  0.91  0.93  1.00 | --  0.87-0.96  0.88-0.97  0.95-1.05 | --  0.001  0.002  0.942 | --  1.03  0.97  0.95 | --  0.96-1.10  0.92-1.02  0.90-1.01 | --  0.421  0.234  0.105 |

ERCP ,endoscopic retrograde cholangiopancreatography
