# Supplementary material for: Racial differences in primary sclerosing cholangitis: A retrospective cohort study leveraging a new ICD-10 code
Source: Ann Hepatol. Author manuscript; Available in PMC 2026 Mar 11. (PMC12826390; doi:10.1016/j.aohep.2025.101901)
Supplement: Supp 5 [file NIHMS2089701-supplement-Supp_5.docx]

**Supplemental Table 1.** Covariate ICD codes

| Diagnosis | ICD-9 Code | ICD-10 Code |
| --- | --- | --- |
| Sepsis | 785.52, 995.92 | A021, A227, A267, A327, A400, A401, A403, A408, A409, A410.1, A410.2, A411, A412, A414, A413, A415.0, A415.1, A415.2, A415.3, A415.9, A418.1, A418.9, A419, A427, A548.6, R652.0, R652.1 |
| Bacteremia | 790.7, 038.0, 038.1, 038.10, 038.11, 038.12, 038.19, 038.2, 038.2, 038.4, 038.40, 038.41, 038.42, 038.43, 038.44, 0.38.49, 038.8, 038.9 | R7881 |
| Pancreatitis | 577.0 | K85, K85.1, K82 |
| Diabetes | 50, 49 | E10, E11 |
| Hypertension | 401.0, 401.1, 401.9, 405.0, 405.01, 405.09, 405.11, 405.19, 405.9, 405.91, 405,99 | I10, III, I12, I13, I14, I15, I16 |
| Cholestatic Disease | 574.0, 574.00, 574.01, 574.1, 574.10, 574.11, 574.2, 574.20, 574.21, 574.3, 574.30, 574.31, 574.4, 574.40, 574.41, 574.5, 574.50, 574.51, 574.6, 574.60, 574.61, 574.7, 574.70, 574.71, 574.8, 574.80, 574.81, 574.9, 574.90, 574.91, 575.0, 575.1, 575.10, 575.11, 575.12, 575.2, 575.3, 575.4, 575.5, 575.6, 575.8, 575.9 | K80, K80.0, K80.00, K80.01, K80.1, K80.10, K80.11, K80.12, K80.13, K80.18, K80.19, K80.2, K80.20, K80.21, K80.3, K80.30, K80.31, K80.32, K80.33, K80.34, K80.35, K80.36, K80.37, K80.4, K80.40, K80.41, K80.42, K80.43, K80.44, K80.45, K80.46, K80.47, K80.5, K80.50, K80.51, K80.6, K80.60, K80.61, K80.62, K80.63, K80.64, K80.65, K80.66, K80.67, K80.7, K80.70, K80.71, K80.8, K80.80, K80.81, K81, K81.0, K81.1, K81.2, K81,9 |
| Liver, biliary, and pancreatic malignancy | 155.0, 155.1, 155.2, 156.0, 156.1, 156.2, 156.8, 156.9, 157.0, 157.1, 157.2, 157.3, 157.4, 157.8, 157.9 | C22, C22.0, C22.1, C22.2, C22.3, C22.4, C22.7, C22.8, C22.9, C23, C24, C24.0, C24.1, C24.8, C24.9, C25, C25.0, C25.1, C25.3, C25.4, C25.7, C25.8, C25.9 |
